# First PCR Confirmed anthrax outbreaks in Ethiopia—Amhara region, 2018–2019

**DOI:** 10.1371/journal.pntd.0010181

**Published:** 2022-02-10

**Authors:** Baye Ashenefe Wassie, Surafel Fantaw, Yonas Mekonene, Amete Mihret Teshale, Yohannis Yitagesu, Estifanos Tsige, Desalegn Getahun, Geremew Tasew, Getachew Abichu, Beyene Moges, Ebba Abate, Takele Abayneh, Taye Zeru, Zewdu Belay, Siobhan M. Mor

**Affiliations:** 1 Ethiopian Public Health Institute, Addis Ababa, Ethiopia; 2 National Veterinary Institute, Bishoftu, Ethiopia; 3 Amhara Public Health Institute, Bahir Dar, Ethiopia; 4 Amhara Livestock Resource Development and Promotion Agency, Bahir Dar, Ethiopia; 5 Institute of Infection, Veterinary and Ecological Sciences, University of Liverpool, Liverpool, United Kingdom; 6 International Livestock Research Institute, Addis Ababa, Ethiopia; Sibirskij gosudarstvennyj medicinskij universitet, RUSSIAN FEDERATION

## Abstract

**Background:**

Anthrax is a disease that affects humans and animals. In Ethiopia, anthrax is a reportable disease and assumed to be endemic, although laboratory confirmation has not been routinely performed until recently. We describe the findings from the investigation of two outbreaks in Amhara region.

**Methods:**

Following reports of suspected outbreaks in Wag Hamra zone (Outbreak 1) and South Gondar zone (Outbreak 2), multi-sectoral teams involving both animal and public health officials were deployed to investigate and establish control programs. A suspect case was defined as: sudden death with rapid bloating or bleeding from orifice(s) with unclotted blood (animals); and signs compatible with cutaneous, ingestion, or inhalation anthrax ≤7 days after exposure to a suspect animal (humans). Suspect human cases were interviewed using a standard questionnaire. Samples were collected from humans with suspected anthrax (Outbreak 1 and Outbreak 2) as well as dried meat of suspect animal cases (Outbreak 2). A case was confirmed if a positive test was returned using real-time polymerase chain reaction (qPCR).

**Results:**

In Outbreak 1, a total of 49 cows died due to suspected anthrax and 22 humans developed symptoms consistent with cutaneous anthrax (40% attack rate), two of whom died due to suspected ingestion anthrax. Three people were confirmed to have anthrax by qPCR. In Outbreak 2, anthrax was suspected to have caused the deaths of two livestock animals and one human. Subsequent investigation revealed 18 suspected cases of cutaneous anthrax in humans (27% attack rate). None of the 12 human samples collected tested positive, however, a swab taken from the dried meat of one animal case (goat) was positive by qPCR.

**Conclusion:**

We report the first qPCR-confirmed outbreaks of anthrax in Ethiopia. Both outbreaks were controlled through active case finding, carcass management, ring vaccination of livestock, training of health professionals and outreach with livestock owners. Human and animal health authorities should work together using a One Health approach to improve case reporting and vaccine coverage.

## Author summary

Anthrax is a bacterial disease that causes rapid death in livestock, particularly cattle. Humans can be infected when they touch or eat animal carcasses or animal products, or when they inhale bacterial spores that have been aerosolized from the environment. Depending on how the bacterium enters the body, it causes cutaneous, gastrointestinal/oropharyngeal or respiratory disease in humans; death is rare for cutaneous anthrax but is more common with ingestion and inhalation forms of the disease. Anthrax has a global distribution but some areas of the world–such as Ethiopia–experience annual outbreaks in animals and humans. In this report, we present the findings from the first PCR-confirmed outbreaks in Ethiopia. In both outbreaks, the lack of farmer awareness and inherent economic value of livestock led farmers to salvage carcasses of animals that died, for meat and hides. Community members who participated in skinning and butchering of carcasses, eating or handling the meat, or processing hides/skins of suspect animal cases developed clinical signs consistent with anthrax (22 humans in Outbreak 1 including two suspected deaths; and 18 in Outbreak 2 including one suspected death). Further, animal parts from index cases (one cow in Outbreak 1; and one cow and one goat from Outbreak 2) were improperly discarded around the environment, leading a further 48 cows to become infected and die in Outbreak 1. Three humans in Outbreak 1 tested positive for anthrax on PCR. In addition, a swab taken from the dried meat of an animal that died in Outbreak 2 was positive by PCR. In both outbreaks, control was enacted through recalling and disposing remaining animal products, vaccination of livestock in surrounding areas and community education.

## Introduction

Anthrax is an acute infectious disease caused by infection with the spore-forming bacterium, *Bacillus anthracis*. The disease affects almost all warm-blooded animals, including humans [1]. Animals become infected through ingestion of spores in the soil. The disease is rapidly fatal in herbivores such as cattle, with some animals displaying characteristic signs of external bleeding from the nose, mouth and anus upon death. On exposure to air, bacteria released in blood and body fluids form environmentally-resistant spores that can persist for decades in the soil [1]. Humans become infected incidentally through contact with diseased animals or contaminated animal products. In most (95%) cases, exposure occurs via cuts or abrasions in the skin following handling of infected animal products, such as hides or wool. This results in cutaneous lesions characterized by a raised vesicle that develops into a painless sore with black center (‘black eschar’) [1]. Less commonly, ingestion of undercooked meat can lead to oropharyngeal or gastrointestinal disease with nausea, vomiting, abdominal pain and severe bloody diarrhea. Inhalation of spores can also lead to the respiratory form, characterized by flu-like symptoms, difficulty breathing and shock. Whereas treatment of cutaneous anthrax with antibiotics leads to recovery, ingestion and inhalation anthrax are often fatal.

Anthrax has a global distribution with an estimated 1.83 billion people and 1.1 billion livestock living in regions at risk for the disease [2]. Sporadic epizootics in livestock and wild ungulates occur across Eurasia, Africa and North America [2]. An estimated 20,000 to 100,000 cases of anthrax occur in humans worldwide every year [3], mostly among the rural poor, and particularly in sub-Saharan Africa as well as East and South Asia where livestock vaccination rates are very low and used principally to control rather than prevent outbreaks [2]. The global distribution of anthrax is largely determined by the presence of soils that support spore survival, namely those that are rich in organic matter and calcium and with pH>6 [4]. Landscape suitability is further delineated by factors such as livestock density, wild ungulate species richness, forest loss, rainfall and temperature [5–8]. Anthrax seasons are typically characterized by hot-dry weather, with outbreaks often triggered by weather extremes such as heavy rain following a period of prolonged drought [4,9]. As such, a warming climate is predicted to increase anthrax risk in some areas of the world [6].

In Ethiopia, sporadic anthrax outbreaks occur annually among livestock [10], posing health risks to people who come into contact with infected animals. Whilst the disease is recognized by Ethiopian farmers as one of the most important livestock diseases [11] and it is ranked second only to rabies according to the recent joint-ministerial zoonotic disease prioritization exercise [12], Ethiopia has lacked laboratory capacity to safely confirm anthrax in humans and animals until recently. Reports of anthrax in the scientific literature in Ethiopia have therefore been limited to a small number of clinical case reports or series which relied on clinical diagnosis of humans presenting to hospital [13–16]. To our knowledge, only one study, published in 2004, has documented a laboratory-confirmed case (goat) in Ethiopia using microbiological methods [17].

In 2018, laboratory capacity for diagnosis using real-time polymerase chain reaction (qPCR) was established at the Ethiopian Public Health Institute (EPHI) government laboratory in the capital, Addis Ababa. In this paper we present the findings from the epidemiologic investigation of the first PCR-confirmed outbreaks of anthrax in Ethiopia. Both outbreaks occurred in remote areas of Amhara region in northern Ethiopia.

## Materials and methods

### Ethics statement

Procedures for outbreak investigations of anthrax were reviewed and approved by the Institutional Review Board at EPHI (protocol number 070–2017). Written consent was obtained for blood and swab sample collection. Where a suspect case involved a minor, written parental/guardian consent and assent were obtained.

### Location

Amhara region (9° to 13° 45’ N and 36° to 40° 30’ E) is one of 9 divisions of Ethiopia, and is located in the northern part of the country, bordered by the state of Sudan to the northwest. The region is subdivided into 11 administrative zones. Approximately 170,752 sq km in area, Amhara comprises highlands (>2300 m altitude), semi-highlands (1500–2300 m) and lowlands (500–1500 m). Climate varies across the region, ranging between cool/subtropical in the highlands to tropical in the lowlands. Peak rainfall occurs from June to September, with annual rainfall ranging from 510 mm to 200 mm. Mean temperatures ranging from 15–21°C, with the hottest month being May. At the last census (2007), the human population of Amhara region was 17,214,056, with more than 85% of the population residing in rural areas [18]. The population is predominantly Orthodox Christian (82.5%), with approximately 17% and 0.2% identifying as Muslim and Protestant, respectively. Poverty rates, child mortality and malnutrition are amongst the highest in the country [19]. A large majority of households engage in animal husbandry, with an estimated 14.7 million cattle, 10.0 million sheep, and 6.0 million goats farmed in the region [20].

### Epidemiological investigation

#### Outbreak 1

**Fig 1:**
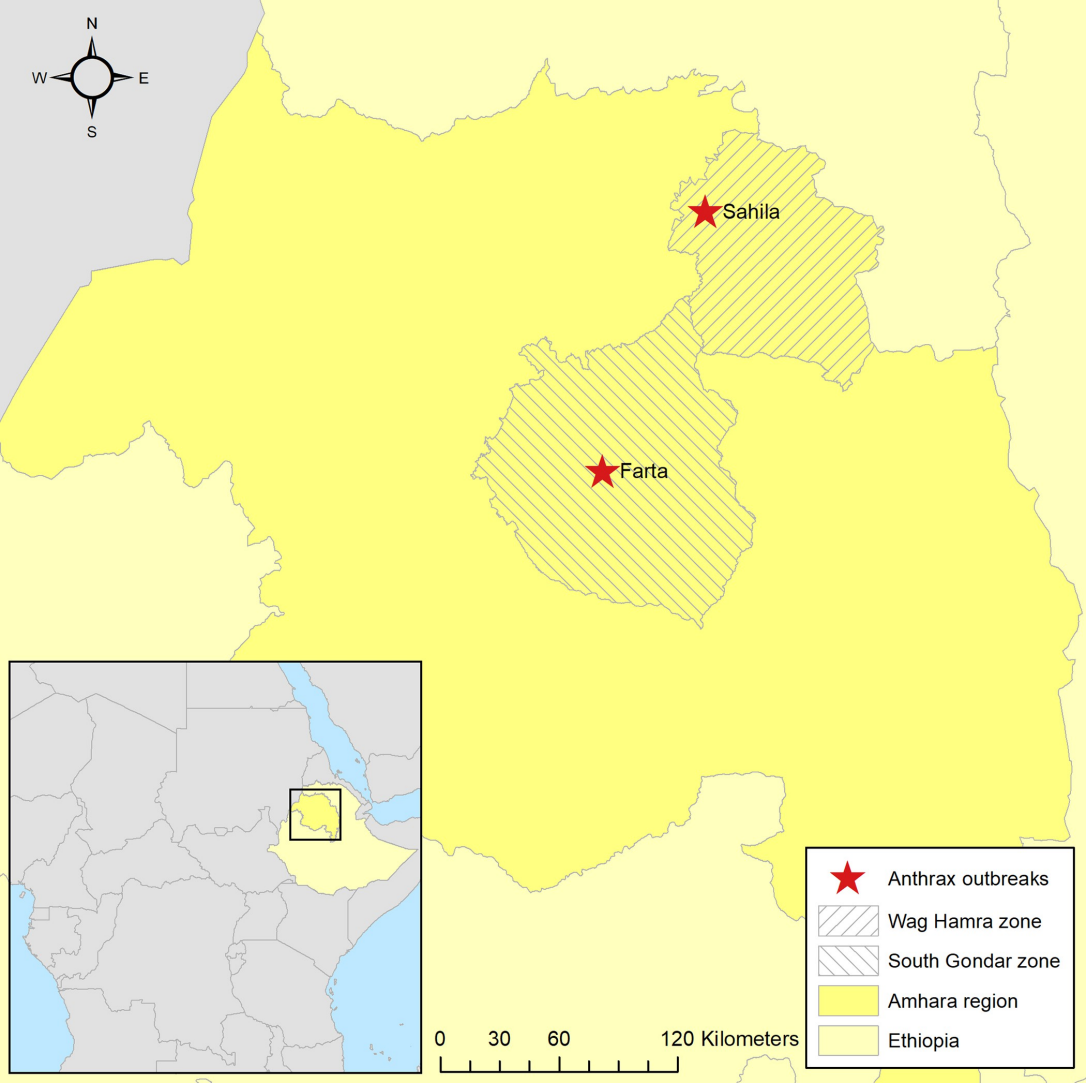
Map of Amhara region in northern Ethiopia, showing locations of anthrax outbreaks in Sahila village, Wag Hamra zone (Outbreak 1) and Farta village, South Gondar zone (Outbreak 2). Inset, map showing Ethiopia within continent of Africa. Base layers for the maps are accessible at https://data.humdata.org/dataset/ethiopia-cod-ab and https://public.opendatasoft.com/explore/dataset/world-administrative-boundaries/.

A suspected outbreak of anthrax in animals in Sahila, a remote village in Wag Hamra zone (Fig 1), was reported to the Amhara Livestock Resource Development and Promotion Agency (ALRDPA) on 24 January 2019. The index case was a cow that died within 12 hours of showing signs of respiratory distress on the evening of 5 December 2018 [non-fasting season]. The outbreak began after a period of rain, which is thought to have brought spores to the soil surface where they could be ingested by ruminants grazing in the area. The first case of anthrax in humans was reported by district health authorities to the Amhara Region Public Health Institute (ARPHI) three days after the death of the index animal case, on 8 December 2018.

National authorities (EPHI and the anthrax technical working group) were first notified of the suspected outbreak by ALRDPA/ARPHI on 21 March 2019. The next day a multisectoral team involving both animal and public health officials from federal and regional sectors was deployed to the location. The team visited the area on 24 March 2019 to investigate the outbreak and establish a control program. Following an interview with the owner of the index cow, a list of all community members who had come in contact with the animal’s meat was generated. Case-finding was initiated through visiting households; the GPS location was recorded and households were mapped to enable follow-up. Enhanced surveillance was initiated on 25 March 2019. A standard questionnaire was used to gather information on demographics, exposures and clinical signs from suspected cases. A case of anthrax was suspected if an animal had signs consistent with anthrax, namely: sudden death with rapid bloating or bleeding from orifice(s) with unclotted blood. In humans, a suspected case was defined as a person with signs compatible with cutaneous, ingestion, or inhalation anthrax ≤7 days after exposure to a suspect animal. A (human or animal) case was confirmed if a positive result was returned on qPCR testing (see below). Specimens were collected from humans with lesions/freshly intact vesicles using sterile swab(s) without removing the eschar and placed in bacterial transport media. Blood samples were also collected in plain vacuum tubes using venipuncture. Primary sample containers were placed in a secondary leak proof container with sufficient absorbent material to absorb any liquid contents in the event of a spill or breakage. Personal protective equipment (PPE) including gloves, disposable gown, shoe covers, eye goggles and face shield was used during sample collection. Samples were transported to EPHI for analysis and stored at recommended temperatures prior to testing.

#### Outbreak 2

A second suspected outbreak of anthrax was reported to EPHI in Farta, a remote village in South Gondar zone (Fig 1), on 9 March 2019. In this outbreak the index animal cases were a cow and goat that died within two days of showing non-specific signs on 7 March 2019 [fasting season]. At the time of notification there was one suspected human death which presented with a history of a painless sore with black center and edema on the face, arms and legs as well as abdominal pain, nausea, vomiting and bloody diarrhea in the six days preceding death. As for Outbreak 1, a multisectoral team from national and regional health and livestock organizations visited the area on 10 March 2019 to investigate the outbreak and establish a control program. Following an interview with the owner of the animals, a list comprising all people who came in contact with the animal’s meat and carcass was generated and case-finding was initiated as for Outbreak 1. Enhanced surveillance was initiated on 25 March 2019 accordingly, using the case definitions above. A standard questionnaire was used to gather information from suspected cases. Samples were collected from humans as detailed for Outbreak 1. In addition, up to two swabs were collected from the dried meat and hide of suspect animals and placed in lavender top (EDTA) vacutainers. All samples were triple packed, transported and stored as described for Outbreak 1.

### Laboratory investigation

Testing was carried out in the EPHI clinical microbiology laboratories in Addis Ababa. Spores were recovered from swabs using the swab extraction tube system (SETS; Roche Applied Science, Indianapolis, IN). Swabs were rehydrated in 500 μl PBS in the outer tubes, transferred to the inner tube and then centrifuged for 1 min at 10,000 x g to collect the eluted cell suspensions in the outer tubes as previously described [21]. DNA was subsequently extracted from eluted swab cell suspensions and serum samples using Qiagen DNA minikit and Qiagen DNA blood minikit (Qiagen Inc., Valencia, CA), respectively, according to manufacturer’s directions. Primers and probe sets targeting genes encoding protective antigen (*pagA*) and capsular protein B (*capB*) were used (Table 1). These targets are located on virulence plasmid pX01 and pX02, respectively, and distinguish from non-virulent *Bacillus* spp. which lack these plasmids. Two quality controls spiked with *pagA* and *capB* targets were included as positive controls in the extraction step, as was nuclease-free water as a blank control. For the qPCR, primers and probes targeting the human *RNase P* (RNP) gene were also included as an internal control. Reactions were prepared with: 1 μl of each primer and probe (final concentration 0.2 μM); 12.5 μl PerfeCTa Multiplex qPCR Supermix, Low ROX (Quantabio Inc., Beverly, MA); 2.5 μl 10X Exo IPC mix (Applied Biosystems); 0.5 μl 50X Exo IPC DNA (Applied Biosystems); 1.5 μl nuclease-free H_2_O; and 5 μl of template, for a total reaction volume of 25 μl. Amplification was performed on an ABI 7500 fast real-time PCR system (Applied Biosystems, Foster City, CA) as follows: 95°C for 1 min (optimization) followed by 40 cycles of 95°C for 10 s (denaturation), 55°C for 30 s (annealing and extension). Samples positive (Ct<35) for all three targets (*pagA*, *capB* and RNP) were deemed positive for anthrax. All clinical specimens and consumables were autoclaved prior to disposal.

**Table 1:** Primer and probes used to detect *B*. *anthracis* in two outbreaks of anthrax in Amhara region, northern Ethiopia, 2018-2019. Primers were supplied by the United States Centers for Disease Control and Prevention.

Primer/probe	Sequence (5’-3’)	Target gene	Reference
PA-forward	CGG ATC AAG TAT ATG GGA ATA TAG CAA	*pagA*	[50]
PA-reverse	CCG GTT TAG TCG TTT CTA ATG GAT	*pagA*	[50]
PA-probe	6FAM-CTC GAA CTG GAG TGA AGT GTT ACC GCA AAT-BHQ1	*pagA*	[50]
CAP-forward	ACG TAT GGT GTT TCA AGA TTC ATG	*capB*	[50]
CAP-reverse	ATT TTC GTC TCA TTC TAC CTC ACC	*capB*	[50]
CAP-probe	6FAM-CCA CGG AAT TCA AAA ATC TCA AAT GGC AT-BHQ1	*capB*	[50]
RNP-forward	AGA TTT GGA CCT GCG AGC G	*RNase P*	[51]
RNP-reverse	GAG CGG CTG TCT CCA CAA GT	*RNase P*	[51]
RNP-probe	6FAM-TTC TGA CCT GAA GGC TCT GCG CG-BHQ1	*RNase P*	[51]

Probes were labeled at the 5’-end with the reporter molecule 6-carboxyfluorescein (6FAM) and at the 3’- end with the quencher, Black Hole Quencher-1 (BHQ1).

### Statistical analysis

Attack rates were calculated as the number of suspect or confirmed cases divided by the number of people exposed to suspect animal carcasses. Similarly, case fatality rates were calculated as the number of suspected deaths divided by the number of people with suspected anthrax. Attack rates were stratified by age and gender and odds ratios were calculated. Fisher’s exact test was used to compare attack rates between groups. Vaccine coverage in livestock was estimated as the number of vaccinated animals divided by the total number of livestock in the area.

## Results

### Outbreak 1

**Fig 2:**
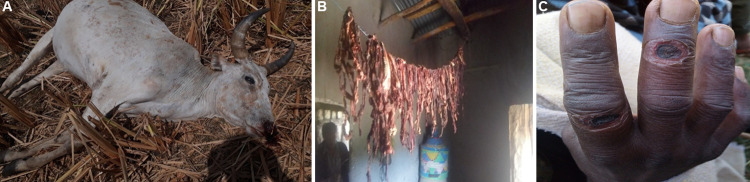
Photographs taken during outbreaks of anthrax in Amhara region, northern Ethiopia, 2018–2019. A, deceased cow displaying typical bleeding from the nose in Sahila village, Wag Hamra zone (Outbreak 1); B, dried (goat) meat that tested qPCR-positive in Farta village, South Gondar zone (Outbreak 2); C, cutanteous lesions (‘black eschars’) on qPCR-positive case in Farta village, South Gondar zone (Outbreak 2).

Upon investigation, it was found that the owner of the index animal case had opened the carcass and dressed the meat, which he shared or sold within the village for human consumption. The hide, bones and offal were disposed of around the environment, including in water bodies. Contamination of the environment with products from the index animal case resulted in the exposure and death of other animals within proximity to the same village. Affected animals were observed by the owner and animal health experts to have unclotted blood oozing from the orifices (Fig 2A). In total, 49 cows (including the index case) died with suspected anthrax during the outbreak period (5 December 2018–24 January 2019). Further, two dogs and one cat died during the same period following a brief illness (vomiting, diarrhea and breathing difficulties) and were possibly exposed to *B*. *anthracis* through ingestion of meat, bones and offal from infected carcasses. No samples were collected from suspect animal cases as meat had been consumed and carcasses had been disposed by the time of the multi-sectoral outbreak investigation.

In total, 55 persons in the village (including the owner) came into direct contact with the infected carcass of the index cow through the skinning/butchering process or by consuming the meat, handling the meat/hide or disposing animal products, many without using any protective clothing or equipment. Twenty-two people developed painless, vesicular cutaneous lesions with itching sensation, typical of anthrax, on the arm, finger, leg or back, resulting in an overall attack rate of 40%. The highest attack rate was observed in people aged ≥41years, with comparatively lower attack rates in younger people (Table 2; p = 0.054). Higher attack rates were observed in women, although this was not statistically significant (Table 2; OR = 1.29 [95% CI: 0.42, 3.9], p = 0.65). Two cases (females aged 16 and 35 years) were suspected to have died due to the disease, resulting in a case fatality rate of 9%; both fatal cases manifested clinically as cutaneous and ingestion anthrax, although this diagnosis was not confirmed as post-mortem samples were not taken. Blood samples were collected from 13 people with suspected cutaneous anthrax, of which three were found to be positive on qPCR. None of the four swab samples collected from humans with cutaneous lesions tested positive for anthrax. All people with suspected cutaneous anthrax were treated with doxycycline and were subsequently cured. Fortunately, given heightened community awareness of the situation, no humans contracted anthrax from the secondary animal cases.

**Table 2:** Incidence of cutaneous anthrax in humans, by age and sex, in two outbreaks of Amhara region, northern Ethiopia 2018-2019. Numbers include two cases in Outbreak 1 and one case in Outbreak 2 that were suspected to have died due to the disease; all fatal cases manifested clinically as cutaneous and ingestion anthrax. OR = odds ratio, CI = confidence interval.

Demographic characteristics	Outbreak 1 (Sahila village, Wag Hamra zone)					Outbreak 2 (Farta village, South Gondar zone)				
	Cases	Exposed population	Attack rate per 100	OR (95% CI)	P-value	Cases	Exposed population	Attack rate per 100	OR (95% CI)	P-value
Age, years					0.054					0.760
0–20	6	13	46	1		6	17	35	1	
20–40	9	32	28	0.46 (0.12, 1.73)		9	36	25	0.61 (0.18, 2.13)	
≥41	7	10	70	2.72 (0.48, 15.47)		3	13	23	0.55 (0.11, 2.80)	
Gender					0.653					0.721
Female	14	33	42	1.29 (0.42, 3.9)		10	39	26	0.82 (0.27, 2.45)	
Male	8	22	36	1		8	27	30	1	

On confirmation of the outbreak, the Animal Resources Development Office of the government of Wag Hamra zone immediately carried out prevention and control measures, including ring vaccination of animals in surrounding villages. A post-outbreak campaign involving administration of 24,772 vaccines to the 383,038 livestock in the area, resulted in a vaccination coverage rate of 35% (up from 28% prior to the outbreak). During the outbreak response, the team held meetings to make village residents and students aware of the risks associated with anthrax and requesting that animal movement be restricted to the nearest grazing areas. In total, 180 male and 65 female livestock owners were educated about associated risk factors and public health implications of anthrax.

### Outbreak 2

Upon investigation, it was found that the carcass of the cow and goat was shared by the community. As it was the fasting season, meat was dried for later consumption while the hide, bones and offal were disposed of around the environment, including in water bodies. In this outbreak, however, only two animals died during the outbreak period (7 March– 28 March 2019). Swabs collected from the dried meat of one of the animal cases (goat) (Fig 2B) were found to be positive by qPCR.

In total, 66 people came in direct contact with an index animal case during the skinning and butchering process or through handling meat, of whom 18 developed cutaneous lesions that are compatible with anthrax (overall attack rate = 27%; Fig 2C). Of the 18 suspected human cases, ten were females and eight were males; there were no clear patterns in terms of sex and age (Table 2). No further human deaths were reported during the period of enhanced surveillance. Samples were collected from 12 people with suspected cutaneous anthrax, however none of the six blood samples or six swab samples tested positive for *B*. *anthracis* on qPCR.

Control was enacted by recalling all animal products from confirmed and suspected animal cases followed by disinfection and destruction by burning as per World Health Organization (WHO) guidelines. Similar to Outbreak 1, animal ring vaccination was initiated and on-site capacity building for health and veterinary professionals was undertaken as was as a community roadshow with distribution of communication materials. A post-outbreak campaign involving administration of 275,349 vaccines to the 413,023 livestock in the area, resulted in a vaccination coverage rate of 78% (up from 12% prior to the outbreak).

## Discussion

In this paper we document two outbreaks of anthrax in northern Ethiopia. Although the disease has long been suspected to be endemic, these outbreaks represent the first PCR-confirmed outbreaks of anthrax in the country. Until now, laboratory confirmation of suspected anthrax outbreaks has not been routinely performed in Ethiopia due to lack of facilities to safely undertake culture and identification from clinical specimens. Establishing capacity for qPCR at the national public health institute laboratory has contributed to improved outbreak detection and patient management, through greater engagement of national authorities in outbreak response. While the features of these outbreaks are similar to others in the African literature, this paper is, to our knowledge, the first to report the detection of *B*. *anthracis* DNA in swabs taken from dried meat.

In both outbreaks, inappropriate carcass management had been practiced by affected communities resulting in human and animal cases. Although we did not investigate community knowledge, attitudes and practices, a recent study in Wag Hamra zone (location of Outbreak 1 in this paper) found that the majority (98%) of farmers knew of anthrax and many (two thirds) identified vaccination and burial/burning of carcasses as important preventive measures [22]. Nonetheless, around three quarters of farmers in that study still reported consuming meat and using skins and hides from animals suspected to have died from anthrax; food insecurity was cited by the authors as the major reason for adopting such practices. This has been reported in other African countries and reflects the desire to salvage the economic value of the animal as well as poor awareness of the communities [23–27]. Because sporulation occurs upon exposure to air, the World Organisation for Animal Health (OIE) advises that carcasses of animals that are suspected to have died from anthrax should, in fact, not be opened; the putrefaction process kills vegetative forms of the bacteria inside the carcass [28]. As an extra precaution, because of the potential for environmental contamination and persistence of spores in the soil, most countries recommend that carcasses be burned, buried and/or treated with formaldehyde [1]. When this is not feasible, as may be the case in low income countries, carcasses should be covered with tarpaulins, tree branches or other available materials to prevent access by scavengers [1].

In Outbreak 2, we detected *B*. *anthracis* DNA in swabs taken from the dried meat of one of the index animal cases (goat). This finding contributes to the literature on the utility of dried animal products as a useful substrate for anthrax detection in low income countries when other samples, such as blood, are not available at the time of investigation [29]. Due to the extensive practice of fasting in Ethiopia–in which observers of the Ethiopian Orthodox faith abstain from consumption of all animal products for around 180 days each year–meat is frequently dried for later consumption in the non-fasting season. This may have contributed to the relatively small number of deaths related to ingestion anthrax as meat was not immediately consumed by the community. Whether the dried meat was still infectious at the time of sampling could not be ascertained as only molecular detection was employed. To our knowledge, the only other report of *B*. *anthracis* detection in dried meat is from an outbreak on an Indian (Native American) reservation [30]. In that outbreak, *B*. *anthracis* was detected in sun-dried beef (jerky) using laboratory culture and immunofluorescence; the timing of sample collection relative to the outbreak onset was not stated in that investigation.

Unfortunately, due to problems with the swab sample taken from hide in Outbreak 2, we were not able to perform PCR on this specimen. However, the fact that *B*. *anthracis* can persist and remain infectious on dried hides is widely acknowledged, with a number of outbreaks in North America and Europe linked to making and/or playing drums made from animal skins and hides [31–37]. Given the expanding national and international market for skin and hides in Ethiopia [38], there may be opportunities to link anthrax prevention activities to broader initiatives aimed at growing the leather industry in the country. In particular, producers and collectors of raw skins and hides need to be trained on the risks of anthrax and advised not to process animals that are suspected to have died from anthrax.

In Outbreak 1 deaths among dogs and cats during the outbreak period were noted following a period of illness. These animals were potentially exposed to infected carcasses and were presumed to have died due to anthrax, however, lack of laboratory testing of these suspect cases makes this difficult to confirm or refute. Carnivores, including domestic dogs (*Canis lupus familiaris*), are thought to be relatively resistant to anthrax [39]. Nonetheless, deaths have been reported when domestic dogs were fed meat and bones from livestock that died of anthrax [40,41]. A high anthrax seroprevalence has been recorded in domestic dogs following outbreaks in wildlife in the Serengeti ecosystem of Tanzania [42] as well as in multiple areas of Zimbabwe [43], suggesting frequent, non-fatal exposure in these domestic animals. Similar findings have been reported in wild carnivores, with lions (*Panthera leo*), spotted hyenas (*Crocuta crocuta*) and/or jackals (*Canis mesomelas*) showing high seroprevalence but few deaths in the Serengeti ecosystem [42], Etosha National Park in Namibia [44] and Zimbabwe [45]. In contrast, anthrax-related deaths have been reported amongst cheetahs (*Acinonyx jubatus*) in Botswana [46] and African wild dogs (*Lycaon pictus*) in Tanzania [47]. No similar studies of anthrax–in domestic or wild carnivores–have been undertaken in Ethiopia. This may be an important knowledge gap, particularly since Ethiopia is home to one of the rarest of wild canids, namely the highly endangered Ethiopian wolf (*Canis simensis*). Populations of *C*. *simensis* have been decimated by outbreaks of rabies and canine distemper virus; their susceptibility to anthrax remains unknown [48].

This study has some important limitations. As mentioned, the delays in reporting and subsequent investigation of Outbreak 1 (>100 days) meant that samples from animals (cattle, dog and cat) were not available for laboratory testing. Further, sampling of older cutaneous lesions in people may have contributed to false negatives. The refusal of some community members to divulge information and consent to sample collection also meant that data were incomplete. Finally, no secondary test was undertaken to confirm the qPCR results owing to the lack of suitable laboratory facilities for culture and identification from clinical specimens (ideally biosafety level 3; [1]) in Ethiopia. Nonetheless, according to WHO “Genetically-based confirmation by [PCR] is becoming increasingly accepted on a stand-alone basis for many types of specimen and is increasingly available worldwide through commercial kits” [1]. This includes in the United States where the Council for State and Territorial Epidemiologists considers PCR sufficient confirmation in a case meeting the clinical criteria [49].

In conclusion, we report the first PCR-confirmed outbreaks of anthrax in Ethiopia. Inappropriate carcass management by the community contributed to high attack rates and some fatalities. Better surveillance and reporting, with strengthened One Health coordination is needed for early identification of outbreaks and is crucial in prevention and control of this disease. Awareness raising campaigns with the community as well as onsite training among human and animal healthcare workers in the field is needed to ensure prompt detection and response to outbreaks.
